# Beneficial or Harmful Role of Macrophages in Guillain-Barré Syndrome and Experimental Autoimmune Neuritis

**DOI:** 10.1155/2018/4286364

**Published:** 2018-04-26

**Authors:** Donghui Shen, Fengna Chu, Yue Lang, Yunlong Geng, Xiangyu Zheng, Jie Zhu, Kangding Liu

**Affiliations:** ^1^Neuroscience Center, Department of Neurology, The First Hospital of Jilin University, Jilin University, Changchun 130021, China; ^2^Department of Neurobiology, Care Sciences and Society, Karolinska Institute, Karolinska University Hospital, SE-14157 Huddinge, Stockholm, Sweden

## Abstract

Guillain-Barré syndrome (GBS), an immune-mediated demyelinating peripheral neuropathy, is characterized by acute weakness of the extremities and areflexia or hyporeflexia. Experimental autoimmune neuritis (EAN) is a common animal model for GBS, which represents a CD4^+^ T cell-mediated inflammatory autoimmune demyelination of the peripheral nervous system (PNS), and is used to investigate the pathogenic mechanism of GBS. It has been found that macrophages play a critical role in the pathogenesis of both GBS and EAN. Macrophages have been primarily classified into two major phenotypes: proinflammatory macrophages (M1) and anti-inflammatory macrophages (M2). The two different macrophage subsets M1 and M2 may play a decisive role in initiation and development of GBS and EAN. However, recently, it has been indicated that the roles of macrophages in immune regulation and autoimmune diseases are more complex than those suggested by a simple M1-M2 dichotomy. Macrophages might exert either inflammatory or anti-inflammatory effect by secreting pro- or anti-inflammatory cytokines, and either inducing the activation of T cells to mediate immune response, resulting in inflammation and demyelination in the PNS, or promoting disease recovery. In this review, we summarize the dual roles of macrophages in GBS and EAN and explore the mechanism of macrophage polarization to provide a potential therapeutic approach for GBS in the future.

## 1. Introduction

Guillain-Barré syndrome (GBS) is an inflammatory demyelinating peripheral neuropathy, characterized by acute weakness of the extremities and areflexia. Acute inflammatory demyelinating polyneuropathy (AIDP) and acute motor axonal neuropathy (AMAN) are the common subtypes of GBS [1, 2]. The pathological features of GBS include inflammation, demyelination, and axonal damage in the peripheral nervous system (PNS) [2]. Currently, intravenous administration of immunoglobulin or plasma exchange is the optimal treatment approach [2, 3]. However, 3–10% of patients with GBS do not survive while 20% live with severe disabilities [4]. Although the pathogenesis of GBS is still unclear, it may be associated with cellular and humoral immune responses [4]. AIDP is related to CD4^+^ T cell-mediated inflammation and macrophage-induced demyelination of the PNS, while AMAN mostly involves the autoantibodies against gangliosides [5]. Experimental autoimmune neuritis (EAN) is an artificially induced demyelinating animal model that mimics the pathological and immunological features of GBS [6, 7]. Similar to GBS, EAN is characterized by activated T cell and macrophage infiltration into the PNS, broken blood-nerve barrier (BNB) and inflammatory demyelination, and axonal injury of the peripheral nerves [4, 7].

Macrophages present different phenotypes and contribute to host immune response, metabolic homeostasis, and tissue repair [8, 9]. Activated macrophages are generally divided into two phenotypes, namely proinflammatory macrophages (M1) and anti-inflammatory macrophages (M2) [10]. Multiple lines of evidence suggest that the macrophages have phenotypes with high plasticity, which could be altered by appropriate signals in different pathological conditions [11, 12]. Macrophages play either a proinflammatory or anti-inflammatory role in the different stages of GBS [7]. M1 macrophages are associated with inflammatory impairment of the myelin sheath by promoting cellular cytotoxicity and production of Th1 cytokines during the early course of GBS [13, 14]. M2 macrophages, in contrast, are involved in the recovery of disease and repair of myelin and axon by facilitating Th2 immune response as well as the secretion of anti-inflammatory cytokines in the later phase of GBS [14, 15]. Increasing studies have demonstrated that the switch of macrophage phenotype from M1 to M2 could effectively ameliorate the severity of EAN [16–18].

## 2. An Overview of Macrophages

Generally, macrophages come from the embryonic progenitors or blood monocytes and exist in various tissues of the body. The phenotypic and functional properties of macrophages are determined by the signaling molecules they encounter in the beginning.

As a remarkably heterogeneous cell type, macrophages present with different names in various tissues of the body, such as microglia in the central nervous system (CNS), Kupffer cells in the liver, red pulp macrophages in the spleen, osteoclasts in the bones, and macrophages in the alveoli [11, 19]. As resident macrophages in the liver, Kupffer cells are located in the periportal area of the sinusoids and are able to endocytose pathogens and apoptotic cells for host defense [20]. Alveolar macrophages are the immune effector cells in the lungs, which can be recruited into the alveolar space to facilitate the clearance of inhaled pathogens and the secretion of inflammatory molecules [21]. Microglia contribute to the maintenance of the neural environment and clearance of the debris and dying neurons in the brain by altering their morphology and releasing different cytokines and mediators that exert proinflammatory or anti-inflammatory roles [22, 23].

Macrophages exert multiple functions, including presenting antigens and eliminating microbes and tumor cells as well as remodeling tissues. Macrophages, with a broad range of pathogen recognition receptors, are implicated in innate and adaptive immune response by phagocytosis and recognition of pathogen-associated molecular patterns [8]. During the immune response, the major histocompatibility complex (MHC) I and II antigens are expressed and upregulated by the macrophages responsible for antigen presentation. Moreover, macrophages participate in the activation of T helper (Th) cells and the production of inflammatory cytokines and chemokines [14]. They also express adhesion molecules, which are involved in the recruitment of lymphocytes into the inflammatory lesions. In addition, macrophages play a neuroprotective role by secreting neurotrophic factors and reducing the inflammatory response [15, 24]. They display a spectrum of functions and phenotypes depending on the numerous cytokines and pathogens they encounter in the microenvironment of the body [19].

Macrophages have been classified into two phenotypes mirroring T cell polarization: proinflammatory macrophages (or M1) and anti-inflammatory macrophages (or M2) [25]. M1 and M2 macrophages represent two extreme states of macrophage activation. M1 macrophages mediate the host defense and secrete proinflammatory cytokines and molecules to cause tissue damage and disease development, whereas M2 macrophages express high levels of anti-inflammatory molecule to reduce inflammation and promote disease recovery [10, 24]. The activation of macrophage, from the resting state to the M1 state, is generally induced by microbial products and proinflammatory cytokines and molecules, such as lipopolysaccharides (LPS), interferon-*γ* (IFN-*γ*), interleukin-1*β* (IL-1*β*), and tumor necrosis factor-*α* (TNF-*α*) [26, 27]. M1 macrophages not only produce high levels of proinflammatory cytokines and molecules, and oxidative metabolites such as nitric oxide (NO) and reactive oxygen intermediates (ROI) [27, 28], but also promote the expression of MHC-II and costimulatory molecules [28]. Moreover, they exert strong microbicidal and tumoricidal activities [10, 28]. IL-4, IL-13, IL-10, and immune complexes (IC) drive the polarization of resting macrophage to the M2 phenotype [10, 27, 28], which supports tissue repair and suppresses destructive immunity. It upregulates the expression of scavenger molecule receptor CD163 and mannose receptor CD206 and increases phagocytic activity for pathogens and apoptotic cells and the synthesis of trophic factors [27]. Activated M2 macrophages contribute to tissue remodeling by producing high levels of IL-10, transforming growth factor-*β* (TGF-*β*), and downregulating proinflammatory cytokine expression [27]. M2 macrophages can be further subcategorized into M2a, M2b, and M2c depending on their inductive stimuli and secreted chemokines, which are involved in the Th2 response, immunoregulatory activities, tissue remodeling, and angiogenesis [29, 30]. Both IL-4 and IL-13 induce M2a polarization, consequently recruiting Th2 cells into the lesions of inflammation or injury, thereby mediating the Th2 response. M2b polarization is induced by immune complexes (IC) and TLR agonists (LPS). M2b exerts immunoregulatory effects and recruits regulatory T (Treg) cells, in addition to inducing their crosstalk with B cells and promoting antigen presentation [31]. The polarization of M2c is driven by IL-10 and characterized by the suppression of immune responses and promotion of tissue remodeling [27]. The heterogeneity of macrophages is summarized in Table 1.

Several studies demonstrated that M2 macrophages predominate during the recovery and the repair process, which can improve the disease outcome. Increasing M2 macrophages in the acute phase is an effective therapeutic strategy after ischemic stroke. The ability that M2 macrophages can infiltrate into injured brain parenchyma through BBB is crucial for neuronal recovery. They mediate anti-inflammatory and adaptive immune response, scavenge debris, and promote angiogenesis, tissue remodeling, and repair by secreting protective remodeling factors and anti-inflammatory molecules [32]. Injecting M2 macrophages into EAE rats significantly inhibited the severity of clinic symptoms. The therapeutic effects of glatiramer acetate (Copaxone), dexamethasone, and IFN-*β* in MS patients may be due to promoting M2 macrophage polarization partly [33]. M2 macrophages may reverse the detrimental effects of M1 macrophages and promote axon regeneration by secreting neurotrophic factors, supporting cellular phagocytosis and Th2 cells differentiation and facilitating SC activation after spinal cord injury (SCI) and during the later phase of EAE or wallerian degeneration [34–36]. Therefore, shifting macrophage phenotypes toward M2 subtype may facilitate repair in SCI and peripheral nerve injury.

The transition of macrophage phenotype in the local environment could regulate the initiation, development, and recovery of autoimmune and inflammatory diseases [37]. The functional diversity of macrophages can be attributed to their ability to respond to different microenvironmental stimuli via diverse pathways. Identifying the polarization of macrophages and the associated pathways is essential to effectively utilize macrophages as therapeutic targets in many human diseases.

## 3. Effector Functions of Macrophages in GBS and EAN

The exact role of macrophages in GBS and EAN is still not well understood. But, ultrastructural studies revealed that EAN is characterized by breakdown of the BNB, infiltration of T cells and macrophages into the PNS, and demyelination [7].

The enhancement of BNB permeability and the migration of circulating inflammatory cells across the BNB are critical steps in the early phase of the disease. Macrophages contribute to this process by regulating the production of cytokines, chemokines, adhesion molecules, NO, and matrix metalloproteinases (MMPs) [7].

As the principal antigen-presenting cells (APC) and effector cells, macrophages play a pivotal role in the pathogenesis of EAN by presenting antigens and promoting the Th1 polarization [38]. Polarized Th1 cells in turn induce the activation of M1. M1 can promote the expression of MHC-II, adhesion molecules, ROI, and inflammatory cytokines, resulting in inflammation, broken BNB, and demyelination [7].

Studies showed that the expression of MHC-II molecule on macrophages was strongly upregulated in the demyelinated peripheral nerves [39]. As mainly MHC-II positive cells, macrophages present specific antigens to T cells and promote T cell polarization within the PNS.

In addition, macrophages can express high levels of adhesion molecules and chemokines to induce leukocyte infiltration into the space around neurons or axons [40]. Intercellular adhesion molecule-1 (ICAM-1), a kind of adhesion molecule, was found to be upregulated on endothelial cells in EAN and in the serum of patients with GBS. It is associated with the recruitment and migration of immune cells into the PNS in EAN/GBS [41]. Injecting antibody (1A-29) against ICAM-1 into EAN rats could reduce the recruitment of macrophages, inhibit interactions between immunocompetent cells, and attenuate the disease severity of EAN [42]. Substantially delayed degradation of neurofilament protein and collapse of axonal profiles have been found in the distal nerve segment of ICAM-1^−/−^ mice. The authors proposed that the absence of ICAM-1 could have impaired axonal degeneration and regeneration in the injured peripheral nerves [43]. ICAM-1 can be induced by inflammatory cytokines such as IFN-*γ*, IL-1*β*, and TNF-*α*, which are expressed by macrophages in demyelinated nerves, and is expressed by the vascular endothelium, macrophages, and lymphocytes [41]. Another adhesion molecule, involved in the pathogenesis of EAN and expressed by macrophages, is complement receptor 3, which recruits macrophages into the peripheral nerve by interacting with ICAM-1 [44].

Once adhesion is established, chemokines guide the autoimmune cells into the PNS. Simultaneously, chemokines, such as macrophage inflammatory protein 1*α* (MIP1*α*) and monocyte chemoattractive protein 1 (MCP-1), are primarily secreted by macrophages and contribute to the pathological changes in nerve degeneration [45]. Neutralization of MIP-1*α* and MCP-1 delays the onset of EAN and inhibits clinical signs of EAN and macrophage recruitment [15]. Interestingly, the change of MCP-1 levels may regulate the type of immune cells that infiltrate into peripheral nerves of EAN. A high level of MCP-1 favors monocytes and Th1 cell infiltration during disease progression and peak severity, while the low level induces Th2 cell response in recovery phase [46].

Macrophages are involved in the demyelination of EAN/GBS by releasing many proinflammatory cytokines including TNF-*α*, IL-12, and IL-6. IL-12 is produced by macrophages and DCs, and its levels parallel the disease severity in EAN. IL-12 promotes the differentiation of Th1 cells, production of IFN-*γ*, and proliferation of NK and T cells. Elevated levels of IL-12 and its receptors were found on peripheral blood mononuclear cells at the peak of AIDP [47]. Additionally, upregulated IL-6 levels were found in the peripheral nerves of EAN and in the serum and CSF of GBS patients. IL-6 is responsible for BNB disturbance and infiltration of immune cells [47]. Enhanced expression of TNF-*α* in serum and CSF was detected in the patients with GBS and was correlated with disease activity [7, 47]. TNF-*α* mainly from macrophages plays a pathogenic role in EAN/GBS, such as precipitating in the disruption of BNB, and involving in degeneration and demyelination, which was evidenced by application of neutralizing antibodies against TNF-*α* which ameliorates the clinical sign of EAN [48]. Additionally, when TNF-*α*, IL-12, or IL-6 were injected into the healthy peripheral nerves, respectively, these cytokines caused serious inflammation, BNB breakdown, and marked demyelination [15]. It is noteworthy that TNF-*α* can facilitate immune cells across BNB in GBS by increasing MCP-1 and ICAM-1 expression [49]. Thus, macrophages may form a firm adhesion between monocytes and endothelial cells by directly expressing MCP-1 or indirectly inducing the production of inflammatory cytokines.

Except the proinflammatory cytokines, toxic mediators released from macrophages including inflammatory cytokines, NO, and MMPs cause axonal loss and demyelination. MMPs are mainly secreted from T cells and macrophages. Expression of MMPs is increased in the progressive phase of GBS and EAN, and this upregulation is correlated with the breakdown of BNB and the release of TNF-*α* and IL-1*β* in GBS. The MMPs facilitate the migration of inflammatory cells and the demyelination in the PNS [50, 51]. NO is produced by inducible nitric oxide synthase (iNOS) in macrophages under the simulation by IFN-*γ* and TNF-*α* and related to nerve demyelination in the peripheral neuropathies [52]. Therefore, a complex network of cytokines, chemokines, adhesion molecules, NO, and MMPs is mediated by macrophages in GBS.

Besides the hematogenous macrophages invading the myelin or axons, there are a considerable number of resident macrophages within the endoneurium of peripheral nerve. The resident macrophages of the PNS can rapidly respond to nerve injury by phagocytosing myelin and expressing MHC-II molecule before blood-derived monocytes infiltrate the injury site [53].

In contrast, M2 macrophages exert a neuroprotective role in the pathogenicity of EAN [54]. Depletion of macrophages compromises peripheral nerve regeneration [55, 56]. Further evidence shows that injured nerves induce secretion of apolipoprotein E (apoE), nicotinamide adenine dinucleotide phosphate oxidase 2 (Nox2), and collagen VI, which regulate peripheral nerve regeneration by favoring macrophage M2 polarization and expression of high levels of arginase-1 and CD206 [56–58]. M2 macrophages may contribute to the spontaneous remyelination and regeneration of the axon [40, 59] by promoting T cell apoptosis, suppressing inflammatory responses [15], clearing myelin and axonal debris [24], and inducing the secretion of anti-inflammatory cytokines such as IL-10 and TGF-*β* [30]. Macrophages are the primary source of anti-inflammatory cytokines, including TGF-*β* and IL-10, which are involved in the repair of peripheral nerve and found in CSF during the recovery of GBS. TGF-*β*, as an immunosuppressive cytokine, can inhibit the proliferation and activation of T cell and maintain Treg cells [47]. IL-10 inhibits APC function and reduces proinflammatory cytokine production and MHC expression. Additionally, IL-10 can facilitate humoral immune responses and inhibit T cell proliferation [60]. Studies have found that IL-10 and TGF-*β* mRNA are upregulated in peripheral nerve during recovery of GBS/EAN. Additionally, macrophages synthesize other mediators, including lipocortin-1, IL-6, and TNF-*α*, which may have anti-inflammatory effects under certain context. Increased levels of lipocortin-1 were seen in the injured sciatic nerves during the recovery phase of EAN. It is most highly expressed in macrophages and lymphocytes and exerts an immunosuppressive effect. IL-6 can reduce disease severity by supporting Schwann cell (SC) differentiation and myelin and axon repair, although it is elevated and promotes the inflammation during EAN and in GBS patients as mentioned previously [15]. Although more evidence is needed, an anti-inflammatory role of TNF-*α* has been indicated through several studies [47, 48]. Of that note, macrophages might induce the apoptosis of T cells by secreting NO, TNF-*α*, and toxic radicals within the peripheral nerve [15]. Furthermore, MMPs are also related to restoring the integrity of the PNS [50].

M2 macrophages express abundant arginase (a competitive enzyme of iNOS) and activin A, which induce arginase-1 expression and inhibit the expression of iNOS, resulting in less production of NO. M2 macrophages also contribute to the axonal regeneration and remyelination by secreting growth and differentiation factors. They foster SC proliferation and axonal growth for peripheral nerve repair [61] by releasing the nerve growth factor (NGF) and laminin protein [62]. Oxidized galectin-1 (GAL-1/Ox) produced by SCs promotes axonal regeneration by stimulating macrophages to produce axonal growth-promoting factor [63]. Macrophages are essential for the regeneration of peripheral nerve, removal of debris, and induction of SC proliferation and factor secretion. They exert these effects by secreting growth and differentiation factors and remodeling the extracellular matrix components [61].

Thus, the different subsets of macrophages with multiple roles, including phagocytosis, antigen presentation, and lymphocyte activation, contribute to both axonal damage and demyelination, as well as remyelination and tissue repair at different stages of the EAN [15]. The functional phenotypes of macrophages are altered depending on the local microenvironment.

Macrophage-mediated nerve destruction is the pathological hallmark of GBS [64]. In AIDP, two potential immunological mechanisms for macrophage-mediated invasion of nerves have been proposed. According to the first hypothesis, macrophages infiltrate into the basement membrane of the peripheral nerve and target the antigens on the surface of SC or myelin sheath for inducing SC injury by activated CD4^+^ T cells and inflammatory mediators [65–67]. Additionally, the inflammatory mediators, such as MMPs, or toxic nitric and oxide radicals, are synthesized and released by macrophages and boost the injury of SC and invasion of the myelin sheath. An alternative hypothesis proposes that antibodies may induce macrophages to the myelin or axonal sites of antigen binding and enhance phagocytosis of macrophages by Fc/complement receptors in antibody-dependent macrophage cytotoxicity or by activating the complement-dependent manner [68, 69]. Complement deposition on the outer surface of SC and elevated complement levels in serum and CSF have been found in the AIDP form GBS. It causes the early vesicular changes of myelin and the accumulation of macrophages and demyelination [70]. The pathological features of AMAN differ from AIDP. AMAN is characterized by axon dysfunctions with little demyelination by anti-GM1 antibodies and complement-mediated attack on the axolemma of the nodes of Ranvier [71]. Macrophages infiltrate into the periaxonal space between SC axolemma and the axon, leaving the myelin sheath intact [72].

**Figure 1:**
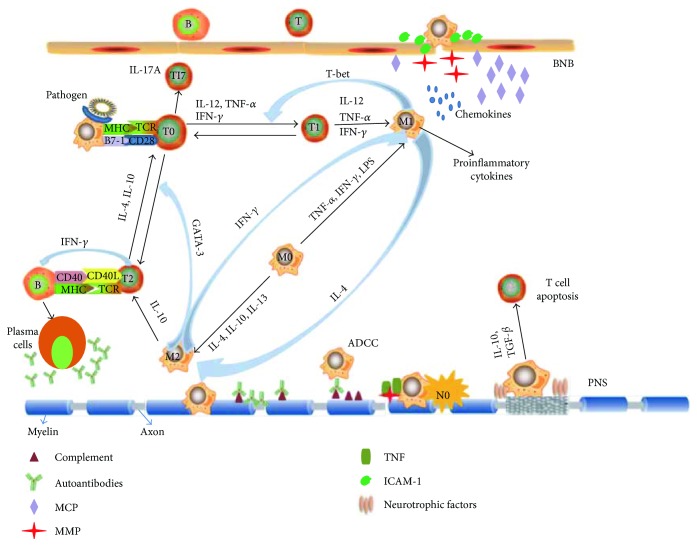
Role of macrophages in the pathogenesis of EAN. Macrophages as the main professional antigen-presenting cells (APCs) by expressing MHC and costimulatory B7-molecules promote the polarization of T cells. Polarized Th1 cells drive macrophages to express a proinflammatory phenotype (M1). Proinflammatory cytokines from M1 macrophages in turn promote the Th1 responses via GATA-3 transcription, while anti-inflammatory cytokines from M2 macrophages facilitate the Th2 response via T-bet transcription. During disease progression of EAN, M1 macrophages contribute to breakdown the blood-nerve barrier (BNB) by releasing adhesion molecules, matrix metalloproteases (MMPs), and chemokines. Macrophages can directly attack myelin or indirectly cause demyelination by antibody-dependent cellular cytotoxicity (ADCC) or complement-dependent manner and releasing proinflammatory cytokines. In addition, nerve destruction occurs through nitric oxide (NO), MMPs, and other cytotoxic radicals. In the recovery phase of EAN, M2 macrophages contribute to remyelination and tissue repair through secreting anti-inflammatory cytokines such as IL-10 and tumor growth factor (TGF-*β*) and promoting T cell apoptosis.

In summary, macrophages not only contribute to the initiation and development of demyelination in GBS by boosting inflammatory events in the PNS, but also play a neuroprotective role by suppressing inflammation, eliminating debris, and promoting PNS repair in the course of EAN and GBS (Figure 1). These properties of macrophages may provide a potential target for the treatment of GBS.

## 4. Transition of Macrophages into M2 Phenotype Ameliorates the Outcome of EAN

Diverse transcription factors, such as signal transducers and activators of transcription 1/6 (STAT1/STAT6), interferon-regulatory factor (IRFs), and peroxisome proliferator-activated receptor-*γ*/*δ* (PPAR-*γ*/*δ*), can regulate M1/M2 polarization programs by interacting with the exogenous and endogenous cellular signaling pathways in the microenvironment [73]. Binding of IFN-*γ* to the cell surface receptor can promote microbicidal activity and proinflammatory cytokine production via the Janus kinase/signal transducer and activator of transcription (JAK/STAT) pathway [27, 74, 75]. It has been recognized that JAK/STAT signaling is essential in the production of IL-1*β*, IL-6, IL-12, IL-23, and iNOS and phosphorylation of STAT1 and STAT3 [27, 76, 77]. In contrast, STAT6 responds to IL-4 and IL-13 and to induce the polarization of M1 subtype [78, 79]. IRF protein may regulate the macrophage polarization. IRF3 and IRF4 contribute to the polarization of anti-inflammatory subtype, while IRF5 is associated with proinflammatory macrophage polarization [80–82]. The transcription factor peroxisome proliferator-activated receptor-*γ*/*δ* (PPAR-*γ*/PPAR-*δ*) can be activated by STAT6, which induces the polarization of macrophages to M2 subtype [27]. Another transcriptional regulator KLF4 cooperates with STAT6 to drive polarization to M2 by sequestering coactivators of nuclear transcription factor-*κ*B (NF-*κ*B) [83].

**Figure 2:**
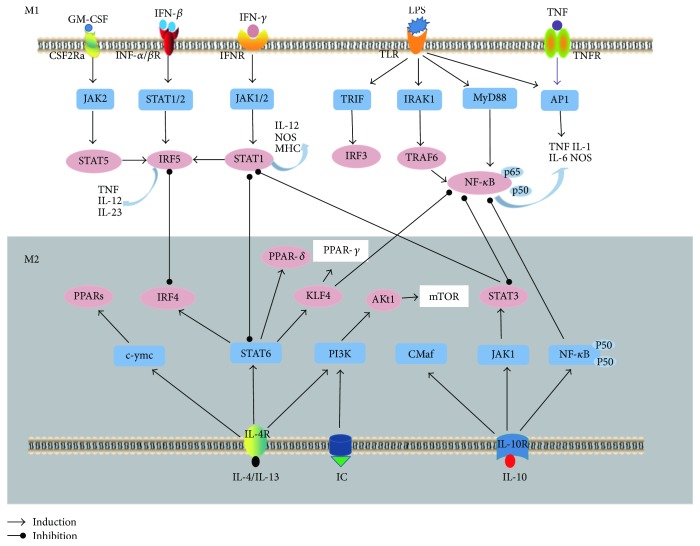
Major signaling pathways involved in M1/M2 polarization. M1 macrophage polarization is mainly induced by granulocyte-macrophage colony-stimulating factor (GM-CSF), interferon (IFN), lipopolysaccharide (LPS), and tumor necrosis factor (TNF), which activate the Janus Kinase/signal transducer and activator of transcription/myeloid differentiation factor 88/nuclear transcription factor-*κ*B (JAK/STAT/MyD88/NF-*κ*B) signaling pathways, which lead to the production of proinflammatory molecules such as inducible nitric oxide synthase (iNOS), TNF-*α*, interleukin- (IL-) 1, and IL-6. IL-4, IL-10, IL-13, and immune complexes (IC) induce M2 macrophage polarization by activating STAT6 and phosphatidylinositide 3-kinases (PI3K) signaling pathways resulting in the upregulation of peroxisome proliferator-activated receptor- (PPAR-) *δ*/*γ* and anti-inflammatory cytokines (IL-10 and tumor growth factor (TGF)-*β*).

As a mitogen-activated protein kinase (MAPK), c-Jun N-terminal kinase (JNK) is associated with cell proliferation, transformation, differentiation, and apoptosis by phosphorylating and deactivating STAT6 [84]. The phosphatidylinositol-3-kinase (PI3K)/Akt signaling pathway activates mammalian target of rapamycin (mTOR) resulting in the promotion of anti-inflammatory macrophage polarization [85]. The signaling pathways involved in M1/M2 polarization are presented in Figure 2. In summary, the present findings suggest that many factors are implicated in the phenotypic and functional switch of macrophages, but the mechanism of involvement of these factors in GBS requires further studies.

The M2 macrophages exert a protective effect against EAN and ameliorate the outcome of EAN by suppressing neuroinflammation and promoting PNS repair [40, 86]. Alternatively, activated M2 macrophages can delay the onset of the clinical symptom and reduce the severity of EAN. Thus, manipulating the signaling pathways to induce macrophage polarization to M2 phenotype may prove to be an effective therapeutic strategy for GBS. Increasing studies have revealed that a variety of substances can regulate the proinflammatory/anti-inflammatory subtype polarization status in experimental animal models. For instance, compound A, as a ligand of glucocorticoid receptors from plant origin, can favor the outcome of EAN by increasing anti-inflammatory macrophages [86]. It has been demonstrated that dimethyl fumarate (DMF) can exert neuroprotective effects by switching Th1 immune response to Th2-dependent immune response, increasing the levels of anti-inflammatory cytokines such as IL-10 and IL-4, suppressing the activity of NF-*κ*B transcription factor, decreasing circulating lymphocytes, and promoting the apoptosis of activated T cells in MS and EAE [87]. Furthermore, DMF also exerts an antioxidant action by increasing the expression of antioxidant enzymes via nuclear factor erythroid-derived factor 2-related factor 2- (Nrf2-) dependent intracellular pathways [88]. It has been reported that Nrf2 exerts an anti-inflammatory and antioxidation effects by inhibiting the transcription of NAD(P)H:quinone oxidoreductase 1 (NQO1) and HO-1 [89]. HO-1 is involved in a protective mechanism against inflammatory responses and oxidative injury by affecting macrophage polarization toward M2 phenotype [90]. Han et al. have reported that DMF suppresses the infiltration of inflammatory cells and demyelination in sciatic nerves by upregulating the level of heme oxygenase-1 (HO-1) and Nrf2 to induce macrophages to polarize toward the M2 type in EAN rats [17].

The mammalian target of rapamycin (mTOR) inhibitor RAD001 (everolimus) alleviates the symptoms of EAN and reduces the inflammatory response by an Akt-mediated phenotypic shift in macrophages to M2 phenotype. RAD001 also increases the production of anti-inflammatory cytokines IL-4 and TGF-*β* in the spleens of EAN rats [18]. Recently, our study revealed that Bowman-Birk inhibitor concentrate (BBIC), a soybean-derived protease inhibitor, decreases the autoimmune response and severity of EAN by inhibiting the transformation and proliferation of macrophages and T cells. In addition, BBIC promotes macrophage polarization to M2 subtype and upregulates the expression of anti-inflammatory cytokines while downregulating the level of proinflammatory cytokines in the PNS of EAN. BBIC might induce M2 polarization by directly or indirectly increasing IL-10 expression [16].

Understanding the dual role of macrophages and the regulatory pathways, in physiological and pathological conditions, may offer novel therapeutic strategies for treating GBS. Further studies are required to further elucidate the therapeutic benefits of modulating macrophage phenotype for treating GBS.

## 5. Conclusion

Although we understand that macrophages play both detrimental and beneficial roles in the pathologies of GBS and EAN, the role of macrophages in an autoimmune disease, such as GBS, is more complex than that suggested by a simple M1-M2 dichotomy. A number of questions are yet to be answered regarding how macrophages are recruited to injury states, how macrophages are involved in PNS degeneration and regeneration, how to shift the polarization of macrophages toward M2 phenotype, and how to improve the outcomes of GBS. Thus, future studies are required to investigate the intricate role of macrophages in the pathogenesis and treatment of GBS.

## Figures and Tables

**Table 1:** Macrophages polarize into M1 (proinflammatory) and M2 (anti-inflammatory) phenotypes.

	M1	M2a	M2b	M2c
Stimuli	LPS; IFN-*γ*; TNF-*α*; IL-1*β*; TLR ligands	IL-4; IL-13	IC; LPS	IL-10
Cytokines	IL-1, IL-6, IL-12, IL-23; IL-1*β*; TNF-*α*	IL-10; IL-R*α*	IL-10	IL-10; TGF-*β*
Chemokines	CXCL8-11; CCL2-5; CCL18; CXCL1-3; CXCL6	CCL17;CCL18; CCL22; CCL24	CCL1	CCL16; CCL18
Gene expression	MHC-II; CD40; CD80; CD86; iNOS IL-12^high^ IL-10^low^	Arg-1; CD163; CD206	MHC-II; CD86 IL-10^high^ IL-12^low^	Arg-1; SLAM
Function	Th1 response Microbicidal activity Tumoricidal activity Antigen presentation NO; ROI	Th2 response Allergy Parasitic infection	Th2 response Recruitment of Treg cell immunoregulation	Recruitment of naïve T cells; Immunoregulation; Tissue repair

IFN-*γ*: interferon-*γ*; LPS: lipopolysaccharide; MHC-II: major histocompatibility complex II; IC: immune complexes; NO: nitric oxide; ROI: reactive oxygen intermediates; TGF: transforming growth factor; TLR: toll-like receptor; TNF-*α*: tumor necrosis receptor-*α*; Arg-1: arginase-1; iNOS: inducible nitric oxide synthase; IL: interleukins; SLAM: signaling lymphocytic activation molecule.

## Additional Points

We carried out a comprehensive search of PubMed for articles published using the following search terms: macrophages, Guillain-Barré Syndrome, experimental autoimmune neuritis, and peripheral nervous system. We performed a review of published articles.
